# Comparison of synthetic bone graft ABM/P-15 and allograft on uninstrumented posterior lumbar spine fusion in sheep

**DOI:** 10.1186/s13018-018-1042-4

**Published:** 2019-01-03

**Authors:** Martin G. Axelsen, Søren Overgaard, Stig M. Jespersen, Ming Ding

**Affiliations:** 1 Department of Orthopedic Surgery & Traumatology, Orthopaedic Research Laboratory, Odense University Hospital, J.B. Winsloewsvej 15, 3rd floor, 5000 Odense, Denmark; 2 Department of Clinical Research, University of Southern Denmark, 5000 Odense, Denmark

**Keywords:** Posterolateral spinal fusion, ABM/P-15, Sheep, Micro-CT, Histology

## Abstract

**Background:**

Spinal fusion is a commonly used procedure in spinal surgery. To ensure stable fusion, bone graft materials are used. ABM/P-15 (commercial name i-Factor™ Flex) is an available synthetic bone graft material that has CE approval in Europe. This peptide has been shown to improve bone formation when used in devices with fixation or on bone defects. However, the lack of external stability and large graft size make posterolateral lumbar fusion (PLF) a most challenging grafting procedure. This prospective randomized study was designed to evaluate early spinal fusion rates using an anorganic bovine-derived hydroxyapatite matrix (ABM) combined with a synthetic 15 amino acid sequence (P-15)–ABM/P-15 bone graft, and compared with allograft in an uninstrumented PLF model in sheep. The objective of this study was to assess fusion rates when using ABM/P-15 in uninstrumented posterolateral fusion in sheep.

**Methods:**

Twelve Texas/Gotland mixed breed sheep underwent open PLF at 2 levels L2/L3 and L4/L5 without fixation instruments. The levels were randomized so that sheep received an ABM graft either with or without P15 coating. Sheep were euthanized after 4.5 months and levels were harvested and evaluated with a micro-CT scanner and qualitative histology. Fusion rates were assessed by 2D sections and 3D reconstruction images and fusion was defined as intertransverse bridging.

**Results:**

There was 68% fusion rate in the allograft group and an extensive migration of graft material was noticed with a fusion rate of just 37% in the ABM/P-15 group. Qualitative histology showed positive osteointegration of the material and good correlation to scanning results.

**Conclusions:**

In this PLF fusion model, ABM/P15 demonstrated the ability to migrate when lacking external stability. Due to this migration, reported fusion rates were significantly lower than in the allograft group. The use of ABM/P15 as i-Factor™ Flex may be limited to devices with fixation and bone defects.

## Background

Spinal fusion is a commonly used procedure in spinal surgery worldwide and is indicated in the surgical management of different spinal disorders such as degenerative disorders, pain, tumor, deformity, and trauma [1, 2]. Over the last decade, the number of spinal fusion procedures has increased significantly, and in 2008 more than 400,000 fusions were performed annually in the USA [3]. Between 2001 and 2010, 79% to 86% of total interbody fusions were posterior/transforaminal lumbar fusions [4]; this number is estimated to have increased since 2010 [3].

Spinal fusion is a procedure where bone graft material is used to facilitate novel bone formation between two adjacent vertebral bones. The aim of fusion is to segmentally impair movement and stabilization, and the procedure may be performed with or without instrumentation [5, 6]. Many different approaches have been tried, and posterior, anterior, and interbody fusion between vertebral bodies are commonly used [7, 8]. In this study, a posterolateral lumbar fusion (PLF) model was used. PLF is the most commonly used fusion model and also the most challenging model in regard to novel bone formation and graft properties. This is due to lack of external support in fixating graft material and large defect size for novel bone formation.

To achieve solid bone formation between vertebral bones, graft materials are used. Traditionally, autograft from the iliac crest has been the gold standard, as autograft possesses osteoinductive, osteoconductive, and osteogenic properties [9, 10]. Because of limited availability in harvesting autograft and patient donor site morbidity such as pain and bleeding, using alternative materials garners high interest [10–12]. Allograft is the most often used surrogate graft material today and is considered a gold standard second only to autograft for lumbar fusion. Allograft possesses a conductive property and a partial osteoinductive property but no osteogenic property. This is because of the freezing procedure for storage after harvesting [13]. Literature reporting lumbar fusion rates when using autograft or allograft is inconsistent with a range of 40–93% [14, 15].

New graft materials that resemble today’s gold standard but are without the risks and limitations associated with autograft or allograft are needed, and several composite materials have been investigated. ABM/P-15 is a recently investigated composite material, which consists of anorganic bovine-derived hydroxyapatite matrix (ABM) combined with a synthetic 15 amino acid sequence (P-15). P-15 has an identical sequence as found in the cell-binding domain in collagen type-1 (α-chain) [16]. This composite material has been proven to stimulate bone formation. ABM/P-15 bears osteoconductive and osteoinductive properties [17–19]; its osteoconduction (ABM) occurs by providing a three-dimensional matrix for bone ingrowth and by releasing necessary minerals. Its osteoinduction (P-15) occurs by providing binding site for α2-β1 integrin on the surface of bone forming cells. The binding of α2β1-integrins to P-15 initiates natural intra- and extracellular signaling pathways and induces production of growth factors, bone morphogenic proteins, and cytokines [17, 20].

The potential of ABM/P-15 on bone formation has been previously shown in preclinical and clinical studies. ABM/P-15 induces bone formation comparable to allograft in critical sized defects and implant fixation sheep models [21] and also improves bone formation in rat osteoporotic models [22]. ABM/P-15 has had comparable fusion rates as allograft in an interbody ovine fusion model [23] and in humans [24]. It has gained CE approval in Europe and is used today in humans as i-Factor™. To this point, no studies have evaluated ABM/P-15 in a flex formula in a PLF model.

The aim of this prospective randomized study was to evaluate early spinal fusion rates using ABM/P-15 bone graft compared with allograft in a two-level uninstrumented PLF model in sheep. This preclinical evaluation is essential prior to using the ABM/P-15 graft for PLF in clinic. As described, this model indicates other challenges when compared to other bone grafting models. We hypothesized that ABM/P-15 graft material had similar or improved fusion rates compared with traditional allograft in an ovine uninstrumented PLF model.

## Methods

### Animals

Twelve skeletally mature female Texas/Gotland breed sheep were purchased from local farmer. These sheep were 3–5 years old and had body weight of 56–87 kg. Sheep were chosen for this study as they provide a good model regarding bone remodeling as their bones biomechanically share similarities to human bone [25]. When compared with pigs and dogs, sheep are also both easier to acquire with mature bones and are easier to handle [25, 26].

The sheep were acclimated for a period of 8 weeks before surgery. During the experiment, they were given standard food and hay and were allowed free access to water. Staff from the Biomedicine Laboratory, University of Southern Denmark took care of them and monitored their daily activity normally. Their body weights were recorded monthly.

Allograft was obtained from a euthanized healthy donor sheep and was immediately made into chips under sterile conditions with a bone mill (Ossano Scandinavia ApS, Stockholm, Sweden). The chips were kept in an − 80 °C freezer for 3 months. The size of the chips was between 1 and 3 mm, and had irregular structure, which was verified under microscopy. The synthetic bone graft used was ABM/P-15 as i-Factor™ Flex strip (Cerapedics, Westminster, CO, USA), which was a combination of freeze-dried AMB granule, 50 μm in size, coated with P-15 peptide.

### Study design

A prospective randomized paired design was used. Twelve sheep were included according to a statistical power calculation. Sheep were randomly divided into two groups; one group had ABM/P15 located at level L2-L3 and allograft at L4-L5 (*n* = 6) while the other group had allograft at L2-L3 and ABM/P15 at L4-L5 (*n* = 6). This design was used to eliminate bias that could be caused by any difference in bone formation capacity between levels and to ensure the animals were their own control.

**Fig. 1:**
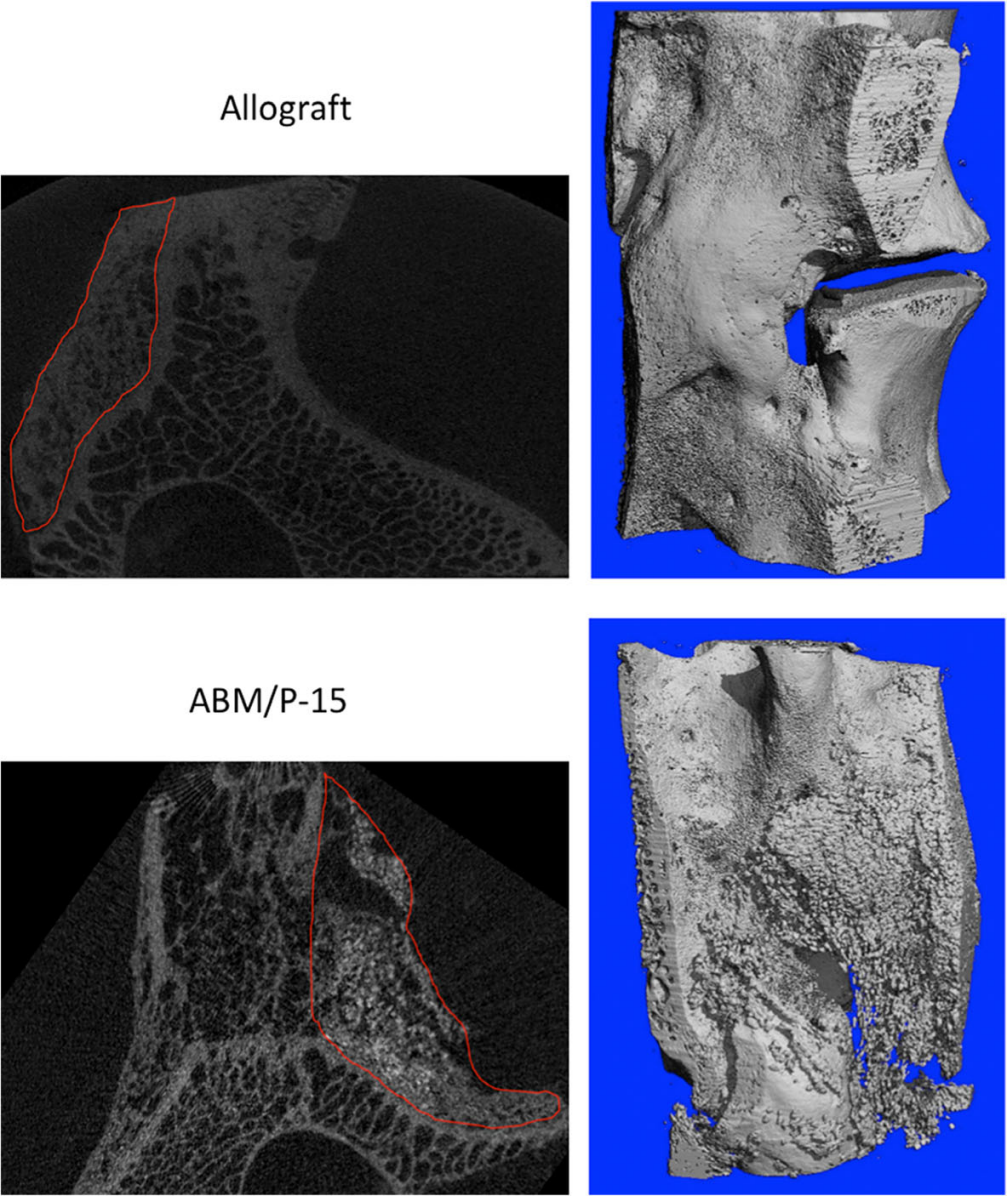
Micro-CT images showed different bone formation patterns: allograft had nice bone formation with a combination of woven and lamellar bones. ABM/P-15 also displayed nice bone formation with clearly visible unresolved residue of hydroxyapatite. Upper left: 2D section of allograft (circle), and upper right: 3D reconstruction of allograft fusion mass. Lower left: 2D section of ABM/P-15 (circle). Upper right: 3D reconstruction of ABM/P-15 fusion mass

All levels were transplanted with same graft material on both sides (Fig. 1). The observation time was set for 4.5 months and was based on our pilot study. Observation time was chosen as fusion with allograft could be expected after this period.

### Surgery

Two days prior to surgery, the sheep were transported to operation facilities to be acclimated. On operation day, the animals were premedicated with Rompun (xylacinhydrochlorid, 20 mg/ml, Bayer animal health GmbH, Leverkusen, Germany) 0.2 mg/kg. Anesthesia was induced with Rapinovent (propofol 10 mg/ml, Schering-Plough animal health, Ballerup, Denmark) 3 mg/kg and maintained with isofloran 2%. Fentanyl 1 mg/kg was given as analgesic during the procedure. The veterinarian at the Biomedicine Laboratory gave the anesthesia and experienced orthopedic spine surgeons performed the surgeries.

The sheep were placed in a prone position, and after shaving and thoroughly disinfecting the area, a posterior access incision was made from lumbar L1 to L6. Dissection was done carefully at level L2-L3 and L4-L5 after identification through palpation from thoracic vertebra 12 with attached costae. There was one level intact (L3-L4) between intervention levels to minimize local interference. Decortication of the transverse processes and opening of the facet joint were performed at L2-L3 and L4-L5. Bone chips from decortication were left at the site at all levels. Both levels were prepared before implantation.

### Graft transplantation

Allograft chips of 5 mg were prepared and weighed in 10 ml syringes. ABM/P-15 was used as i-Factor™ Flex100 and was separated in two; furthermore, 50 mm was used on each side of the same level. After transplantation, the wound was closed in layers.

Postoperatively, all sheep were treated with Temgesic (0.03 mg/ml, Schering-Plough, Ballerup, Denmark) three times daily according to body weight for at least 3 days post-surgery, and treatment lasted no longer than 1 week. Then, 9.0 ml ampicillin (250 mg/ml, Ampivet Vet, Boehringer Ingelheim, Denmark) was given once daily for 5 days. After an observation time of 3–5 days at the animal center, the sheep were moved to farm facilities for further observation until the end of the experiment.

### Sample handling

Sheep were euthanized after 4.5 months with an overdose of 10–20 ml pentobarbital (200 mg/ml), and their spines were harvested. Sample blocks were carefully dissected and soft tissue removed. Macroscopic implant migration was noted. Each vertebral level was divided sagittally through the vertebral body to isolate each implant bilaterally. Samples were then placed in 4% formalin for 3 days and afterward changed into a PBS solution. All blocks were scanned with a micro-CT scanner (detail below) and divided through the middle into two blocks with a sagittal section with EXAKT Diamond Band Saw (Norderstedt, Germany) using a laser light as guide.

### Micro-CT scanning

Micro-CT scanning was performed to validate fusion rates, and fusion was defined as bony bridge formation from two transverse processes. All blocks were scanned with micro-CT50 (Scanco Medical AG, Brüttisellen Switzerland) using energy 90 kV and intensity 155 mA to quantify their 3D microarchitectural properties of the newly formed bone tissue and to discriminate between newly formed bone and implant. The scanned images had 3D reconstruction cubic voxel sizes of 24*24*24 μm^3^ (2048*2048*2048 pixels) with 32-bit-gray-levels. 3D reconstruction was performed and healing was evaluated by 3D images and 2D sections (Fig. 1).

### Histology

Qualitative histology was performed. From scanned images, samples were divided into fusion and non-fusion groups. Randomized samples from each group were prepared for histology by dehydration in graded solutions of ethanol from 70 to 99% and then infiltrated embedded in methyl methacrylate (MMA). Each sample block was divided transversely in the middle using a template to facilitate sectioning. Histological sections were cut sagittally with a custom-made diamond blade Microtome (Medeja Instrumentmakerij, Assendelft, the Netherlands). A random cutoff secured randomization, after which one 50-μm-thick section was dissected from the top, middle, and bottom of the sample and used for qualitative histomorphometry. Sections were stained with toluidine blue 0.1% to differentiate between newly formed bone and mature bone.

### Statistical analysis

Posterolateral lumbar fusion rates assessed by micro-CT at two levels were accessed by chi-squared test using SPSS for Windows, version 25 (SPSS Inc. Chicago, Illinois, USA).

It was planned to perform one-way analysis of variance (ANOVA) to compare the properties among groups. However, due to migration of the ABM/P-15, the planned quantified histomorphometry and microarchitectural analysis were not performed, and statistical analyses were not reported.

## Results

One sheep was euthanized 2 days after surgery as a result of immobilization. Autopsy revealed no nerve damage or other surgical complications and no other complications were noted. In total, 11 sheep completed this study and were used for analysis.

**Fig. 2:**
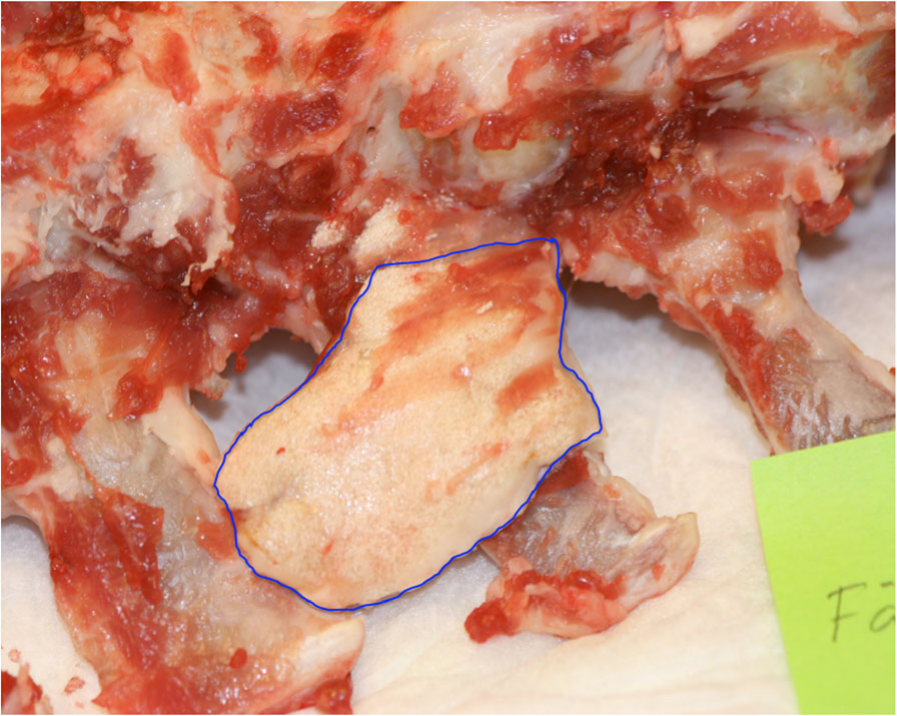
A photo of a migration of ABM/P-15 (blue circle): In this case, migration was ventrally and caudally situated on frontal side of the transverse processes

Spines were harvested after 4.5 months. Macroscopic evaluation revealed migration of ABM/P-15 graft material at all levels. Granules were found either on the ventral side of the transverse processes or had migrated in caudal direction at different degrees. Migrated material was encapsulated and showed no sign of bone formation (Fig. 2). This finding was consistent for all sheep in this study. No migration was found in the allograft group.

For the harvested materials, micro-CT scans were performed and 3D reconstructions were done to evaluate fusion rates. The allograft group had a fusion rate of 68% (Table 1), which was consistent with earlier studies on allograft fusion rates [15, 19]. The ABM/P-15 group showed no complete fusion in connection with bridging of newly formed bone in the transplant (Table 1). Fusion was determined by level that newly formed bone created a stable bridge between transverse processes.

**Table 1:** Posterolateral lumbar fusion rates assessed by micro-CT at two levels

Fusion rates	Fusion	Non fusion	Percentage fusion	Chi-square
Allograft (*N* = 22)	15	7	68%	*P* < 0.01
ABM/P-15 (*N* = 22)	8	14	37%	*P* < 0.01

Eleven sheep with bilateral transplantation on each level (*n* = 22) in both groups

### Histology

**Fig. 3:**
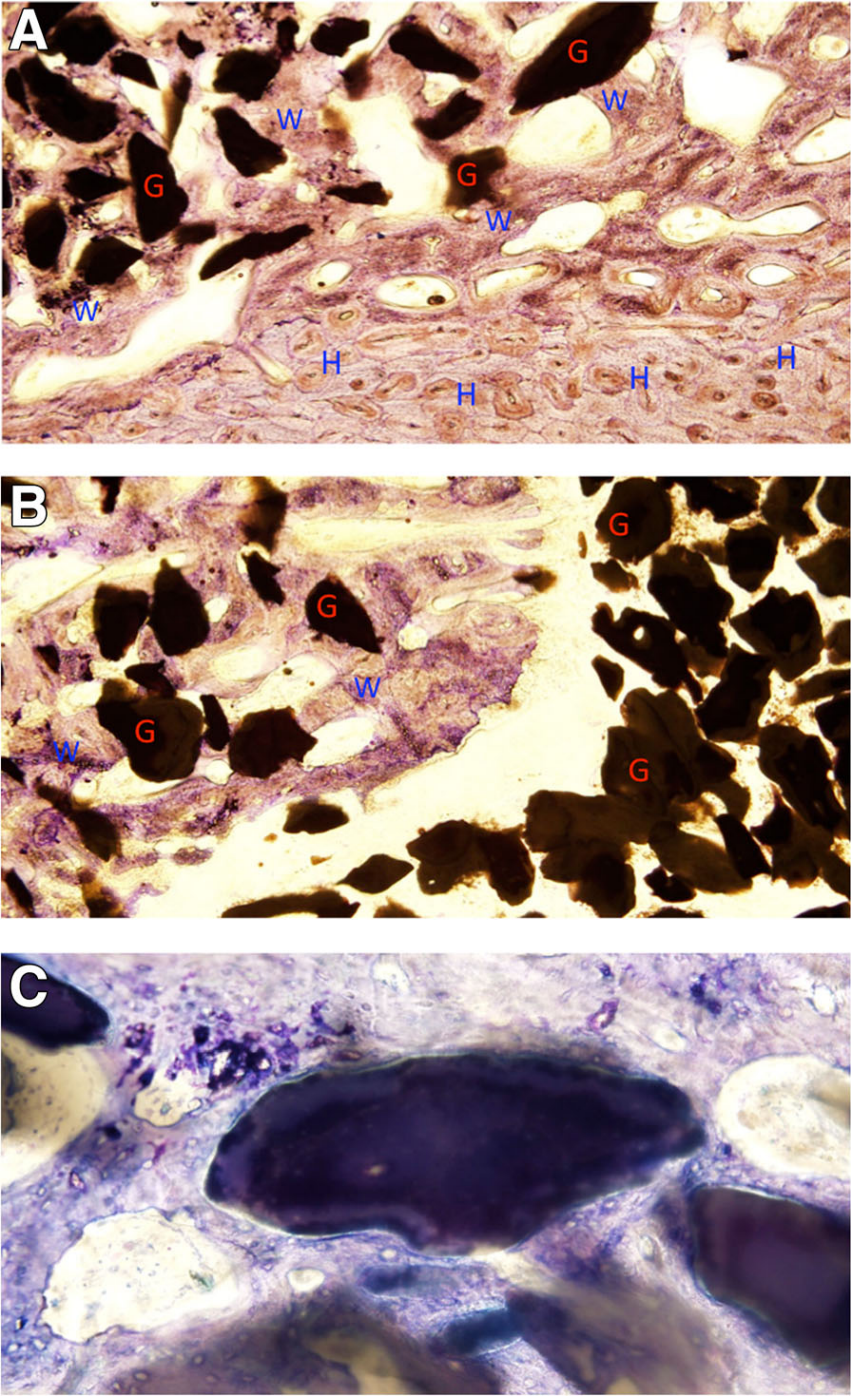
Qualitative histology of fusion section with Toluidine blue 0.1% staining illustrated transition two zones. **a** Proximal transition zone from transverse process (lower-right part) to graft material (upper-left part) is illustrated: cortical bone has typical laminar structure and Haversian canals (H), and ABM granule (G) is surrounded by woven bone. **b** Distal transition zone from graft material (lower-right part) to fibrous tissue (lower-left) in non-fused mass with a clear gap in between. **c** Good osteointegration. Hydroxyapatite granule surrounded by woven bone. No foreign body giant cells

Quantitative histology was performed in both AMB/P-15 and allograft groups. In the ABM/P-15 group, graft material was still evident. New bone formations were found in implant close to the transverse processes in both proximal and distal sections. Good osteointegration between newly formed bone and ABM/-P15 was found and well integrated into pre-existing bone (Fig. 3).

In the ABM/P-15 group, mostly woven bone was present; moreover, few areas showed lamellar initiation. Signs of activity such as osteoid deposition, numerous osteocytes, reabsorption areas, and active surfaces were observed (Fig. 3). There was a well-defined transition zone in the implant between newly formed bone and cartilage (Fig. 3), and no sign of foreign body reaction was found. In the allograft group, graft material was found around mature bone. New bone formation served as a bridge between transverse processes, and new bone formation occurred continuously. Good osteointegration was observed between graft and pre-existing bone, and more areas with lamellar organized bone compared with ABM/P15 group (Fig. 3) were observed.

## Discussion

The aim of this study was to evaluate early spinal fusion rates using ABM/P-15 bone graft compared with allograft in a two-level uninstrumented PLF model in sheep. In the ABM/P-15 group, we found 37% fusion rate while there was 68% fusion rate in the allograft group. Allograft fusion rates are comparable to earlier reported fusion rates in sheep studies [27, 28].

One major cause of failure in the ABM/P-15 group was due to the extensive migration of the graft material. As mentioned earlier, ABM/P-15 bone substitute has been proven to be a suitable bone graft substitute and has gained CE approval in Europe. The bone formation ability was demonstrated when ABM/P-15 was applied in closed containers or in small bone defects, in which settings the surrounding structures supported the implanted bone graft with external fixation.

There are no previous studies that have used ABM/P-15 in this challenging PLF model. The use of ABM/P-15 in this study was comparable with clinical settings and the clinical use of graft material, and is therefore highly clinically relevant [5, 6].

This study has been proven that ABM/P-15 in the i-Factor™ Flex formula migrated when lacking external support as used in an uninstrumented PLF. ABM/P-15 has been approved for human use in Europe and is used today as graft material for spinal surgery; hence, it is vital to make these findings available to surgeons so that they are more aware when using this material in unconfined areas during their procedure. It is expected that improved stability of the material will be required, which means further documentation of its efficacy on spine fusion is needed. Because of migration of ABM/P-15, the planned quantified histomorphometry and microarchitectural analysis were not performed.

The migration rate in the allograft group was not possible to report because allograft material is reabsorbed much faster than ABM-P15. The major component of ABM/P15 is hydroxyapatite and may take 12–24 months to be reabsorbed when migrated [29]. Nevertheless, 68% of bridge formation indicated that sufficient amount of allograft must have stayed at transplantation site. It was a severe mistake that the migrations were found at all the ABM/P-15 transplanted levels. In this study, the graft material was used in a clinically comparable setup and after manufacturer’s guidelines.

The reason for this migration might be found in the smaller size of granule when compared to allograft. When decorticating bleeding was unavoidable, the small size of the granule might have facilitated sedimentation of the granule with blood, which means that it was likely that early migration occurred within the first days after surgery.

Compared with humans, sheep were mobilized faster and were not placed in supine position after surgery. These factors might explain the migration we report in this study. It is thus not directly applicable to humans, and migration might not be as significant a problem as found in this study. It is still a problematic and great concern for clinical application, since migration would cause spinal non-union or delayed fusion.

It is evident that ABM/P-15 as used in this preclinical setup has the ability to achieve major migration. Migration to lesser extent has been reported earlier; in particular, Sherman et al. found migration from cages in an interbody lumbar fusion model [23]. ABM/P-15 bone substitute has been proven to be a suitable bone graft alternative when used in confined containers, devices with fixation, or on small bone defects. It has proven to be a promising bone graft substitute that gives faster and more extensive bone formation when compared to allograft in bone defects [21]. Our next study is to investigate the potential of ABM/P-15 on spinal fusion with improved stability of material.

## Conclusions

Bone substitute ABM/P-15 has been demonstrated to have high potential of migration when used without external fixation in a clinically comparable setting with PLF; and perhaps due to shorter degeneration time, migration of allograft was not found in this study. ABM/P-15 in the i-Factor™ Flex formula revealed significantly lower fusion rates when compared to the allograft group. This finding is important as i-Factor™ Flex has been approved for human use as a bone graft in Europe and is used today in spinal surgery. In humans, migration might be less pronounced due to species differences, which can be seen in slower mobilization and the post-operational supine position of human patients compared to sheep. These findings are important for surgeons who intend to use i-Factor™ Flex in patients, and the material should be used correctly for accurate indications. It is of vital importance to further document the efficacy of i-Factor™ Flex on spine fusion with improved stability of the material.

## Abbreviations

ABM/P-15: An anorganic bovine-derived hydroxyapatite matrix (ABM) combined with a synthetic 15 amino acid sequence (P-15)
PLF: Posterolateral lumbar fusion
